# A randomized controlled trial to evaluate innovative decision support in the context of fall prevention

**DOI:** 10.1038/s41746-025-01822-9

**Published:** 2025-07-11

**Authors:** L. Westerbeek, A. J. Linn, H. C. van Weert, N. van der Velde, S. Medlock, A. Abu-Hanna, J. C. M. van Weert

**Affiliations:** 1 Amsterdam School of Communication Research/ASCoR, University of Amsterdam, Amsterdam, The Netherlands; 2 Amsterdam Public Health Research Institute, Amsterdam, The Netherlands; 3 Department of General Practice, Amsterdam UMC location University of Amsterdam, Amsterdam, The Netherlands; 4 Department of Internal Medicine, Section of Geriatric Medicine, Amsterdam UMC location University of Amsterdam, Amsterdam, The Netherlands; 5 Department of Medical Informatics, Amsterdam UMC location University of Amsterdam, Amsterdam, The Netherlands

**Keywords:** Geriatrics, Randomized controlled trials

## Abstract

Falls are a major cause of injuries among older people, with medication being a key risk factor. The SNOWDROP intervention introduces a clinical decision support system for general practitioners (GPs) offering personalized deprescribing advice, and a patient portal containing information and a question prompt list. This study evaluates the intervention’s effectiveness through a cluster randomized controlled trial in six general practices, with 84 patients (M_age_ = 78.01, SD_age_ = 5.71). Patients discussed their medication-related fall risk with their GP. Data were collected via questionnaires and audio-recorded consultations. The intervention increased shared decision-making for both GPs (*p* < 0.001) and patients (*p* < 0.001), increased patients’ satisfaction with communication (*p* = 0.001), and reduced patients’ decisional conflict (*p* < 0.001). Patients’ beliefs about medication (necessity and concerns) remained stable. The effect on changes to the medication was inconclusive. These results highlight the potential of technology in healthcare and warrant future research.

## Introduction

Technology holds significant potential to enhance the quality of healthcare by making it safer, more efficient, and more effective^1^. This is especially important as the European healthcare sector faces an imminent shortage of personnel^2^ and a high demand, primarily because of the increased prevalence of chronic diseases among an aging population^3^. Furthermore, technology can potentially guide and improve communication processes, such as shared decision-making (SDM)^4,5^. SDM can be defined as a healthcare professional and a patient making healthcare-related decisions together, reflecting both the best available medical evidence and treatment options, and the patient’s personal goals and priorities^6,7^. SDM has increasingly become the new standard in patient-centered care. This is also reflected in the Dutch Medical Treatment Contracts Act (WGBO), which obliges healthcare professionals to decide which care fits best *together* with the patient^8^. However, despite its proven ability to improve the quality of healthcare by increasing patient knowledge, improving health outcomes, and reducing costs, SDM is frequently underutilized in practice^9,10^.

One context in which both technology and SDM can be particularly of great value is preventing medication-related falls among older patients in primary care. As people get older, they often take multiple medications and become more prone to potential side effects^11^. Falling is one of the most prevalent side effects of medication among older adults and can cause a downward spiral, both physically (e.g., reduced mobility) and psychologically (e.g., anxiety). Concerns about falling trigger reduced activity, causing impaired balance and decreased strength, ultimately resulting in a lower quality of life^12^. However, general practitioners (GPs) lack tools to identify at-risk patients and to aggregate the available knowledge on medication and falls. In addition, the patient population in this context consists of older people who often do not feel empowered enough and lack the confidence to engage in SDM^13^.

Technology can increase the efficiency of medication optimization in the context of fall prevention by supporting both GPs and patients^14^. Artificial intelligence (AI) systems use knowledge-based, statistical, or certain other data-driven approaches to generate predictions, recommendations, or decisions^15^. Through the use of AI, advanced decision-support systems have the potential to both identify at-risk patients and provide personalized recommendations based on medical data, supporting the GP in decision-making without overwhelming them with unnecessary complexity. In addition, technology can help identify ways to reduce the fall risk. Medication (i.e., fall risk-increasing drugs; FRIDs) is an important and highly modifiable predictor of falls^16,17^. However, knowledge on these FRIDs is still largely fragmented^18^ and has not been aggregated in a practical tool for GPs. Information about medication-related fall risk for patients with specific health problems is typically embedded in the guidelines for those conditions. This means the knowledge is both complex and fragmented, due to being spread across a large number of guidelines. In this context, a clinical decision-support system (CDSS) has the potential to provide personalized advice to support the GP in the decision-making process. This is particularly valuable when a CDSS is linked to the electronic medical record, as this allows the system to access each patient’s medical record to generate personalized advice without additional work for the GP, removing time constraints as a major barrier for the use of these systems^19^. Finally, patient portals are online systems that patients can access to read and manage their health information, and are increasingly being used to support patients with SDM^20^. These systems can inform and empower patients, resulting in well-prepared patients who are ready to actively participate in the consultation with their GP.

The SNOWDROP intervention (SeNiors empOWered via big Data to joint-manage their medication-related Risk Of falling in Primary care) aims to empower patients and guide GPs to engage in an SDM conversation about medication optimization in relation to the patient’s fall risk. The intervention consists of a CDSS with an integrated prediction model to predict patients’ fall risk, which is embedded in the electronic medical record, and a patient portal. The SNOWDROP intervention is expected to improve SDM by helping the GP to conduct a structured conversation, undertake several SDM steps (e.g., explaining the pros and cons of each option, assessing the patient’s priorities), and by empowering the patient prior to the consultation. Higher SDM scores are associated with higher patient satisfaction^21^, indicating that the intervention may also result in patients being more satisfied with the communication during the consultation. Furthermore, the intervention is expected to reduce the level of decisional conflict experienced by patients; i.e., the extent to which patients experience uncertainty, stress, difficulty in deciding, and worries about undesired outcomes surrounding a medical decision-making process^22^. Research suggests that the amount of decisional conflict experienced by a patient after a medical consultation is often related to inadequate SDM during that consultation^23^. As our intervention is expected to improve SDM between the GP and the patient, it is also expected to reduce the level of decisional conflict experienced by the patient after the consultation. It is hypothesized that the intervention will also influence patients’ beliefs about medication by facilitating a structured discussion about the patient’s current medication, its pros and cons, and possibilities to safely make changes. Finally, because the intervention provides a personalized overview of the options to change a patient’s medication, it is expected that medication changes will be made more frequently and that these changes will be of better quality. The current study aims to evaluate the effects of the SNOWDROP intervention on the communication process, patient-reported outcomes, and medication changes.

## Results

### Sample characteristics

**Fig. 1:**
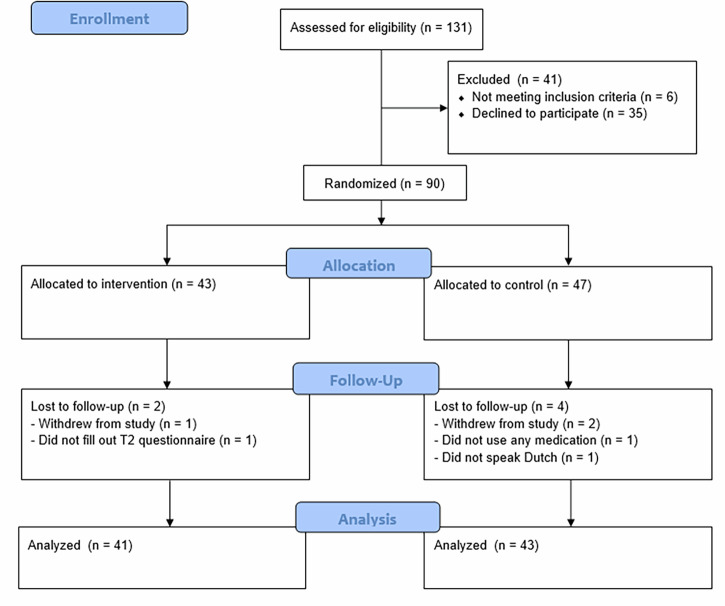
Flowchart for patient inclusion.

Data were collected in six general practices with seven participating GPs between October 2022 and October 2023. Data collection ended when enough patients were included based on the power analysis. Table 1 shows the background characteristics of participating GPs. In total, 131 patients were asked to participate, 90 patients completed the T0 questionnaire, 86 patients had a consultation with their GP at T1, and 84 patients completed the T2 questionnaire. In the end, 84 patients were included in our analyses (see Fig. 1). Please note that the actual number of invited patients might be higher than 131, but is unknown. The number of approached participants was not tracked consistently in one of the participating practices. This could not be checked by the researcher due to privacy regulations. Table 2 presents patient characteristics at baseline for the total sample, as well as for both experimental groups. No significant differences in characteristics between the two groups were found at baseline. Our measurements of decisional conflict (α = 0.92), BMQ necessity (α_T1_ = 0.86, α_T2_ = 0.85), and BMQ concerns (α_T1_ = 0.78, α_T2_ = 0.71) all had an adequate to high reliability. The technology acceptance assessed among intervention GPs and patients had adequate to high reliability for the CDSS (α = 0.79) and the patient portal (α = 0.94). Results showed reasonable acceptance of the CDSS (*M* = 5.06, *SD* = 0.52) and of the patient portal (*M* = 5.23, *SD* = 1.27). A GEE linear analysis showed no significant difference in consultation length (measured in minutes) between the control group (*M* = 21.70, *SD* = 6.51) and the intervention group (*M* = 19.51, *SD* = 6.15), $\beta$ = 1.82, 95% CI [−2.53, 6.17], *p* = 0.412.

**Table 1:** GP characteristics

Variable	Total (*n* = 7)		Intervention (*n* = 4)		Control (*n* = 3)	
Age (years), *M* (*SD*)	53.3	(12.1)	52.3	(14.8)	54.7	(10.1)
Sex (male), *n* (%)	5	(71.4)	4	(100)	1	(33.3)
Years as a GP, *M* (*SD)*	20.0	(12.8)	20.0	(17.7)	20.0	(4.6)
Years as a GP in this practice *M* (*SD*)	16.0	(15.2)	19.5	(18.2)	11.3	(11.7)

*GP* general practitioner.

**Table 2:** Patient characteristics

Variable	Total (*n* = 84)		Intervention (*n* = 41)		Control (*n* = 43)	
Age (years), *M* (*SD*)	78.1	(5.7)	77.3	(5.6)	78.8	(5.7)
Sex (male), *n* (%)	37	(44.0)	20	(48.8)	17	(39.5)
Education level, *n* (%)						
Low	43	(51.2)	19	(46.3)	24	(55.8)
Middle	15	(17.9)	6	(14.6)	9	(20.9)
High	26	(31.0)	16	(39.0)	10	(23.3)
Internet skills, *M* (*SD*)	5.9	(2.4)	6.2	(2.1)	5.5	(2.6)
Health Literacy, *M* (*SD*)	2.3	(0.4)	2.4	(0.3)	2.2	(0.4)

### Inter-observer agreement

The inter-observer agreement of the SDM codes was assessed with an ICC, which was summarized with Cronbach’s alpha. The ICC of the 84 double-coded audio recordings showed high inter-observer agreement for the GPs’ SDM scores (α = 0.91) and for the patients’ SDM scores (α = 0.92).

### Shared decision-making

A GEE analysis with a linear model showed that the intervention had a positive effect on GPs’ SDM scores ($\beta$ = −16.20, 95% CI [−20.77, −11.62], *p* < 0.001). GPs in the intervention group (*M* = 57.84, *SD* = 14.13) scored significantly higher on SDM than GPs in the control group (*M* = 42.94, *SD* = 12.38). A similar effect was anticipated for the SDM scores of patients. The intervention also had a significant effect on patients’ SDM scores ($\beta$ = −11.66, 95% CI [−14.79, −8.53], *p* < 0.001). Patients in the intervention group (*M* = 50.78, *SD* = 15.21) had significantly higher SDM scores than patients in the control group (*M* = 39.62, *SD* = 14.00).

### Satisfaction with communication

Results showed a significant positive effect of the intervention on the satisfaction with the quality of the communication ($\beta$ = −1.20, 95% CI [−1.92, −0.47, *p* = 0.001]). Patients in the intervention group (*M* = 8.80, *SD* = 0.90) were more satisfied with the quality of communication during the consultation than patients in the control group (*M* = 7.47, *SD* = 1.32).

### Decisional conflict

The intervention had a significant effect on the level of decisional conflict experienced by patients ($\beta$ = 10.91, 95% CI [4.99, 16.83], *p* < 0.001). Patients in the intervention group (*M* = 27.40, *SD* = 15.37) experienced significantly less decisional conflict than patients in the control group (*M* = 39.10, *SD* = 9.93).

### Beliefs about medication

Results showed no significant effect of the intervention on patients’ beliefs about medication ($\beta$ = 1.07, 95% CI [−1.19, 3.34], *p* = 0.352). There was no significant difference between patients’ NCD scores in the intervention group (*M* = 4.93, *SD* = 5.16) versus the control group (*M* = 5.52, *SD* = 5.65). We also assessed a possible difference between the T1 and T2 measurements of the NCD, but did not find significant differences in the intervention group (*t* (40) = −0.11, *p* = 0.915) and in the control group (*t* (41) = 0.44, *p* = 0.659).

### Medication changes

There was no significant effect of the intervention on the number of patients for whom one or more medication change(s) were made (OR = 2.09, 95% CI [0.42, 10.40], *p* = 0.366), with no significant difference between the number of patients for whom medication was changed in the intervention group (*M* = 0.56, *SD* = 0.50) versus the control group (*M* = 0.42, *SD* = 0.50). We also saw no significant effect of the intervention on the total number of medication changes made (IRR = 1.23, 95% CI [0.45, 3.41], *p* = 0.684). There was no significant difference between the total amount of changes made in the intervention group (*M* = 0.63, *SD* = 0.66) versus the control group (*M* = 0.77, *SD* = 1.07). In the intervention group, a total of 25 medication changes was made among 23 (out of 41) patients, while in the control group, 33 changes were made among 18 (out of 43) patients. The data showed an outlier among the participating GPs in the control group. This GP made significantly more medication changes than any of the other GPs in both the intervention and the control group, and clearly showed different deprescribing behavior than the other participants (i.e., making 2 or 3 changes for the majority of patients while the others made 0 or 1 change for the majority of their patients). To assess whether this outlier had an influence on the results, the analyses were also conducted without this GP. After removing this GP from the data, the control group consisted of 27 patients, where 10 medication changes were made among 7 patients. Results then showed medication changes were made for a significantly larger number of patients in the intervention group than in the control group (OR = 6.73, 95% CI [1.87, 24.22], *p* = 0.003). The effect of the intervention on the total number of medication changes made is also significant after removing the outlier (IRR = 0.49, 95% CI [0.27, 0.88], *p* = 0.018). The exact fall risk-related medication changes were tracked and grouped into three categories: cardiovascular, psychotropic, and other. When removing the outlier, results show a shift in medication changes from the category other to the category cardiovascular. Results of the other statistical analyses were not affected when removing the outlier GP. Table 3 provides an overview of the types of medication changes that were made, and Table 4 provides a comprehensive overview of all results.

**Table 3:** Overview of medication changes

Medication changed	Control *N* (%)	Intervention *N* (%)	Control without outlier *N* (%)
**Cardiovascular medication**	18 (54.5)	13 (52.0)	1 (11.1)
Thiazide diuretics	3 (9.1)	7 (28.0)	0 (0)
Statins	4 (12.1)	2 (8.0)	0 (0)
Dihydropyridine	3 (9.1)	2 (8.0)	1 (11.1)
Beta-blockers	2 (6.1)	2 (8.0)	0 (0)
Angiotensin receptor blockers	3 (9.1)	0 (0)	0 (0)
ACE inhibitors	2 (6.1)	0 (0)	0 (0)
Cardiac glycosides	1 (3.0)	0 (0)	0 (0)
**Psychotropic medication**	2 (6.1)	0 (0)	2 (22.2)
Tetracyclic antidepressant	1 (3.0)	0 (0)	1 (11.1)
Tricyclic antidepressant	1 (3.0)	0 (0)	1 (11.1)
**Other medication**	13 (39.4)	12 (48.0)	6 (66.7)
Proton pump inhibitors	8 (24.2)	9 (36.0)	2 (22.2)
Alpha-blockers	0 (0)	2 (8.0)	0 (0)
Urinary antispasmodics	2 (6.1)	0 (0)	1 (11.1)
Nonsteroidal anti-inflammatory drugs	0 (0)	1 (4.0)	0 (0)
Antihistamines	1 (3.0)	0 (0)	1 (11.1)
Vertigo medication	1 (3.0)	0 (0)	1 (11.1)
Antiepileptics	1 (3.0)	0 (0)	1 (11.1)

**Table 4:** Results of the GEE analyses

Variable	Control *M* (*SD*)	Intervention *M* (*SD*)	Effect size	95% CI	*p*-value
SDM scores GP-level	42.94 (12.38)	57.84 (14.13)	$\beta$ = −16.20	−20.7, −11.6	<0.001
SDM scores patient-level	39.62 (14.00)	50.78 (15.21)	$\beta$ = −11.66	−14.8, −8.5	<0.001
Satisfaction with communication	7.47 (1.32)	8.80 (0.90)	$\beta$ = −1.20	−1.9, −0.5	0.001
Decisional conflict	39.10 (9.93)	27.40 (15.37)	$\beta$ = 10.91	5.0, 16.8	<0.001
Beliefs about medicines NCD scores	5.52 (5.65)	4.93 (5.16)	$\beta$ = 1.07	−1.2, 3.3	0.352
Nr patients with medication change(s)	*N* = 18 (41.9%)	*N* = 23 (56.1%)	OR = 2.09	0.4, 10.4	0.366
Total number of medication changes	0.77 (1.07)	0.63 (0.66)	IRR = 1.23	0.5, 3.4	0.684
Nr patients with medication change(s) – without outlier	*N* = 6 (22.2%)	*N* = 23 (56.1%)	OR = 6.73	1.9, 24.2	0.003
Total number of medication changes - without outlier	0.33 (0.73)	0.63 (0.66)	IRR = 0.49	0.3, 0.9	0.018

*GEE* generalized estimating equations, *SDM* shared decision-making, *GP* general practitioner, *NCD* necessity-concerns differential.

### Additional analyses

Using GEE with a small number of clusters can inflate the type 1 error rate. Because our sample includes only six clusters, we performed additional cluster-level analyses for all outcome variables to address this concern. Our findings were qualitatively similar, and the conclusions were unchanged when analyzing the data with cluster-level approaches, supporting the robustness of the results.

## Discussion

This study evaluated the effectiveness of the SNOWDROP intervention in a medication-related fall prevention context. Our innovative study using an AI-based, integrated CDSS and a patient portal significantly impacted the communication process. Specifically, patients and GPs who used the intervention engaged in SDM better, compared to patients and GPs in the control group. Patients who had access to the SNOWDROP intervention also experienced less decisional conflict after the consultation and were more satisfied with the communication during the consultation. Beliefs about medication were, however, not affected by the intervention. Results on medication changes were inconclusive.

Our study yielded a marked effect on SDM, with a significant increase in scores for patients as well as for GPs. Previous studies have shown mixed results regarding the influence of different types of interventions on SDM, both in a more general context^9^ and specifically for older patients^24^. This can have multiple explanations. First, interventions targeting SDM are not always developed with their specific end users (e.g., older patients) in mind, resulting in reduced effectiveness^24^. Applying such a user-centered design approach can contribute to the overall effectiveness of an intervention^25,26^. Our intervention was developed specifically for and in close collaboration with our end users (i.e., older patients and GPs), resulting in a system that fits their needs and preferences well. Furthermore, our intervention consisted of multiple components, each targeting SDM in its own way. Multicomponent interventions have been proven to be more effective^27^. The patient portal prompts the patient to think about the topic of the consultation and prepare any questions they might have ahead of time, which might have resulted in a feeling of empowerment. The CDSS, with its embedded prediction model and integration within the electronic medical record, efficiently guided the GP through all of the automatically generated advice and treatment options. This results in a structured conversation that helps them to better address the components of SDM. Additionally, patients in the intervention group were more satisfied with the communication during the consultation than patients in the control group. This is consistent with previous research suggesting that better SDM leads to greater patient satisfaction^21^. Lastly, the intervention group consultations did not take up more time than the control group consultations, refuting the common misconception that SDM is more time-consuming^28^.

The level of decisional conflict experienced by patients in the intervention group was lower than in the control group. High decisional conflict scores experienced after a consultation with a healthcare professional are often the result of insufficient SDM during the consultation^23^. Thus, the reduced levels of decisional conflict found in our study are in line with the increased levels of SDM. The decisional conflict scores found in this study (39.1 control group, 27.4 intervention group) are clinically relevant, even at their lower value in the intervention group. A decisional conflict score of 25 or higher is considered clinically relevant, meaning that the level of decisional conflict is associated with, for example, decisional delay, decision regret, treatment deviation, and nervousness^23,29,30^. Although the intervention was successful in reducing the level of experienced decisional conflict, it still slightly exceeded the cut-off of 25, indicating that there is still room for improvement. In general, changing medication, especially in an older population, causes distress, as patients have often been taking their medication for a long time and may be concerned about possible destabilization of therapeutic effects^31^. It is important to take this into account for future implementation and to look for ways to further reduce the experienced decisional conflict.

Patients’ beliefs about medication did not differ between experimental conditions, nor did they change over time within each condition. Perhaps this is due to the fact that the general topic of the consultations was similar in both conditions (i.e., a medication review focused on fall prevention). It could also be possible that one consultation with the GP is simply not enough to influence beliefs about medication, as these seem to be rather deep-rooted. Research suggests that the BMQ specific (used in this study) is often subject to change over time, depending on medication changes or adverse events^32^, while the BMQ general is more difficult to influence as it measures someone’s general evaluation towards medication use^33^. However, although we used the BMQ specifically, we framed the items in a more general way, covering the patient’s medication use in general, rather than one specific type of medication. Perhaps this more general approach contributed to the lack of an effect on this outcome measure.

The effects on the medication changes made during the consultations remain ambiguous. Initially, there were no significant effects on medication changes. However, one of the GPs included in the control condition was clearly an outlier and made many more medication changes than any of the other participating GPs, showing a vastly different pattern of deprescribing. When this outlier was removed from the data, we found that changes were made for a significantly higher number of patients in the intervention condition than in the control condition, and that the total number of medication changes made was significantly higher in the intervention condition compared to the control condition. This suggests that the intervention has the potential to influence the deprescribing process. The individualized advice automatically generated for each patient seems to be effective in assisting the GP to gain a comprehensive overview of the options for changing medications safely and effectively. The observed difference was not determined by the well-known fall risk-increasing medication group ‘psychotropics’ but by the categories ‘cardiovascular’ and ‘other’ fall risk-increasing medications, suggesting that individualized, guideline-based advice indeed influences the SDM process for these groups specifically. This is promising, as both physicians and patients are most reluctant to change cardiovascular medication, while these changes do contribute to lowering the fall risk^34^. However, it should be noted that this study is underpowered to detect all effects on medication changes, especially after removing the outlier from the data. Thus, although a prompt for a medication review combined with a FRIDs list results in deprescribing of FRIDs, the quality of deprescribing appears to be higher when the CDSS and patient portal are applied. In this small sample, cardiovascular FRID deprescribing and other medication deprescribing were better aligned with the decision rules of the CDSS and thus the underlying (deprescribing) guidelines. To further disentangle the possible effects on medication changes, it is necessary to evaluate this intervention with a larger sample.

The systematic approach taken while carrying out this RCT is a strength of this study. However, carrying out data collection with GPs and patients in their daily practice also comes with some challenges and limitations. First, because of a primary care backlog due to COVID-19 and shortages in personnel, the recruitment of GPs was tough, and many felt they did not have enough time to participate. As a consequence, the sample might have consisted of a group of rather motivated GPs. Therefore, we cannot be certain about the effect of the intervention when making it available to GPs who feel less motivated to use it. Furthermore, not every participating practice consistently tracked which patients declined participation at the first point of contact. This made it impossible for us to assess for potential non-response bias.

The SNOWDROP intervention consists of multiple components, targeting both the GP and the patient. On the one hand, this is an asset, as research has shown that multicomponent interventions tend to improve several aspects of primary care^35^. On the other hand, this does make it difficult to establish which specific components contributed to which effects. Specifically, we know the intervention as a whole influenced the level of SDM, decisional conflict, and satisfaction with the communication during the consultation, but we cannot confirm which parts of our intervention caused these effects. Future research could further explore the effectiveness of this intervention with a larger sample with more power, to be able to distinguish between several components and background characteristics relevant to the intervention.

This study pioneers by integrating AI-based decision support in primary care through a CDSS embedded in the electronic medical record and a patient portal, showcasing its potential to benefit both patients and GPs by enhancing SDM, improving satisfaction with the communication during a consultation, and reducing the level of experienced decisional conflict. Implementation of the intervention in practice has the potential to improve the quality and effectiveness of the decision-making process.

## Methods

### Study design

To evaluate the effectiveness of the SNOWDROP intervention, a cluster randomized controlled trial (RCT) was conducted in six general practices in the Netherlands. The RCT included measurements at three points in time: T0 was a baseline questionnaire prior to the consultation, T1 was an audio recording made during the consultation, and T2 was a follow-up questionnaire two weeks after the consultation. To prevent contamination between experimental conditions, randomization took place at practice level with a 1:1 allocation ratio, resulting in a control arm (*n* = 3 practices, 3 GPs) and an intervention arm (*n* = 3 practices, 4 GPs). Data collection lasted from October 2022 to October 2023. The medical ethical committee of the Amsterdam UMC issued a waiver for this study (W22_114 # 22.154), stating that the Medical Research Involving Human Subjects Act (WMO) does not apply. Ethical approval was then obtained by the Ethics Review Board of the Faculty of Social and Behavioral Sciences, University of Amsterdam (2022-PC-15274). The RCT was preregistered at ClinicalTtrials.gov (ID: NCT05611008). Written informed consent was obtained from all participants (both GPs and patients). The CONSORT statement was followed when reporting this RCT^36^.

### Recruiting general practices and study participants

GPs were actively recruited in several ways. GPs from the Amsterdam UMC Academic Network of General Practice were approached by e-mail or phone, and the trial was presented to their steering committee. Additionally, one of the authors (HW) contacted GPs from outside the network to ensure enough GPs were recruited to include the desired number of patients. A random allocation sequence was generated with an online randomization tool (https://ctrandomization.cancer.gov/) by one of the authors (LW) in the presence of an independent external observer. Randomization took place at the practice level, meaning that the random allocation sequence was used to assign each general practice to either the control or intervention group. Any patient enrolled in the study was automatically assigned to the condition to which their general practice had been allocated.

Patients, in turn, were selected based on their fall risk and medication use. The following inclusion criteria applied: (1) The patient was 65 years or older; (2) The patient had a high fall risk according to our prediction model (i.e., a fall risk of 30% or higher, where the cut-off was based on the average fall risk in the population); and (3) The patient used at least one fall risk-increasing drug (FRID) at the time of inclusion. Patients were excluded if they (1) were cognitively impaired (e.g., had dementia; as assessed or was known by the GP); (2) could not access the internet themselves or with the help of an informal caregiver; or (3) were not able to conduct the survey and consultation in Dutch. Each GP was asked to include about 16 patients. For each practice, a list of eligible patients was created based on data from the electronic medical record. This list was provided to the GPs in a randomized order and was then used from the top down to approach patients until enough patients were included. This was done to avoid possible selection bias that could occur if GPs had selected patients themselves. Patients were not explicitly made aware of the condition they were allocated to, although patients in the intervention group were, of course, aware that they were administered a patient portal.

### Intervention components

The SNOWDROP intervention is summarized below, and described in detail elsewhere (LW, AL, JvW, HvW, AA, SM, 2025, in preparation). The intervention was systematically developed following the Medical Research Council (MRC) guideline for complex interventions^37^.

### Summary of the SNOWDROP intervention

The intervention consists of two components. The first component is a CDSS that guides the clinician. A part of the CDSS is a prediction model that predicts the probability of a fall in the coming twelve months, thereby identifying patients with a high fall risk^42^. The CDSS runs autonomously without user input, as it is integrated within the electronic medical record. The prediction model is used to efficiently and effectively screen patients on their fall risk and identify those who may benefit from preventive measures such as medication changes. The CDSS links patient health data accessed through the electronic medical record with a knowledge base of if-then rules based on over thirty existing guidelines concerning fall risk-increasing medications. The system provides tailored advice to the GP on possibilities to alter a patient’s medication in order to diminish their fall risk. The intervention was accompanied by a short training web lecture (10 min), providing information on how to conduct a medication review focused on the fall risk and how to apply SDM when doing this.

The second component of the intervention is a patient portal tailored to its end users (older people). The patient portal contains general information about falling and fall prevention to ensure the patient feels informed and prepared for their consultation. It also contains a question prompt list (QPL), which is a list of sample questions and/or topics to guide the patient in asking relevant questions during the consultation, thereby empowering older people to engage in SDM^43^.

### Procedure

GPs who decided to participate in the RCT first received a briefing about the procedure of the RCT, during which practical matters such as frequency and process of enrolment were discussed. Intervention GPs were additionally asked to watch an eleven-minute web lecture specifically created for this study, and were informed on how to use the system. In the Netherlands, patients can be invited for a medication review as part of usual care. GPs in the control condition were asked to perform a medication review focused on the patient’s fall risk and received a list of FRIDs on paper. GPs in the intervention condition were asked to perform the same type of consultation, but received support from our CDSS with its integrated prediction model and personalized advice. Patients in the intervention group had access to the patient portal, while control group patients did not.

The general practices always initiated the first contact with the patient. The patients received a phone call and an information letter, either via physical mail or e-mail. In both the intervention and control groups, patients were invited for a consultation to discuss their fall risk in relation to their medication usage with their GP. After indicating that they would be willing to participate and giving permission to share their contact details, the GP shared their contact details with the researcher. The researcher then contacted the patient to answer any questions they might have, obtain informed consent, and distribute the T0 questionnaire. Patients in the intervention condition also obtained access to the patient portal at this point in time and used this to prepare for the consultation, prior to filling out the T0 questionnaire. All patients (both intervention and control) filled out the T0 questionnaire, consisting of background variables, beliefs about medication, and intervention patients also about satisfaction with the patient portal.

The patient then visited their GP for a consultation in which a medication review focused on their fall risk was performed. The researcher was always present in the practice in case of questions and to turn on the audio recording (T1). To minimize the extent to which the RCT influenced the course of the consultations, audio was recorded with a small and discrete recorder, and the researcher was never present in the room during the consultation. Intervention GPs used the CDSS during the consultation. GPs in the intervention condition filled out a questionnaire to evaluate the CDSS after the first and the last consultation. Patients were contacted two weeks after the consultation to fill out the T2 questionnaire, measuring satisfaction with the patient portal, decisional conflict, beliefs about medication, and satisfaction with the quality of communication during the consultation.

### Measurements

Table 5 describes all measurements.

**Table 5:** Overview of measurements

Measurement	Instrumentation
Level of observed SDM (GP)	The adapted Observer OPTION-MCC codebook was used to assess the extent to which GPs engaged in SDM. The level of SDM was scored for each individual consultation (meaning a GP was scored multiple times). The codebook assessed seven items. *Goal talk* concerned identifying patient values and goals of care. *Option talk (1)* involved identifying alternate options and the need for a decision. *Team talk* looked at forming a partnership (i.e., the GP supporting the patient). *Option talk (2)* concerned the GP giving information about options or checking understanding of the options. *Decision talk (1)* looked at eliciting the patient’s preferences, and *decision talk (2)* involved integrating those preferences and making a decision. Lastly, the *evaluation talk* concerned evaluating the SDM process with the patient and formulating the treatment plan. Each item was scored on a scale ranging from 0 (no effort) to 4 (exemplary effort). These scores were later transformed to a score ranging from 0 to 100 by taking the mean score and multiplying it by 25^38^.
Level of observed SDM (patient)	The adapted Observer OPTION-MCC codebook was also used to assess the extent to which patients engaged in SDM. This was also scored for each consultation (meaning each consultation received a GP score and a patient score). The codebook assessed the same seven items on a scale ranging from 0 (no effort) to 4 (exemplary effort) and transformed to a scale ranging from 0 to 100^38^.
Decisional conflict	Decisional conflict was a secondary, patient-reported outcome measured with the Decisional Conflict Scale^30^. This scale consisted of 16 items, such as ‘I knew the benefits of each option’, ‘I made a decision without pressure from others’, and ‘This decision was easy for me to make’. All items were measured on a 5-point Likert scale. The final score was calculated in line with the user manual, by subtracting 1 from the mean score and multiplying by 25, and ranged from 0 (no decisional conflict) to 100 (extremely high decisional conflict)^30^.
Beliefs about medication	The Beliefs About Medicines Questionnaire (BMQ) was used as a secondary outcome to assess patients’ perceived necessity versus concerns regarding their medication^39^. The BMQ consists of 10 items; 5 items about the necessity of medication (necessity subscale; e.g., ‘my life would be impossible without medication’), and 5 items regarding concerns about medication (concerns subscale; e.g., ‘having to take medication worries me’). All items were assessed on a scale ranging from 1 (strongly disagree) to 5 (strongly agree). Scores for each subscale were summed, for instance, meaning a higher score on the concerns scale indicated a patient had more concerns about their medication. To calculate a necessity-concerns differential (NCD), the sum score of the concerns scale was subtracted from the sum score of the necessity scale, resulting in a score between −20 and 20. A higher score on this differential indicates that a patient valued the necessity of their medication higher than their concerns. Items were framed to cover the patient’s medication use in general, rather than one specific type of medication.
Satisfaction with the quality of communication	Satisfaction with the quality of the communication during the consultation was measured with a patient-reported grade ranging from 1 to 10. The question asked was: ‘On a scale from 1 to 10, how satisfied were you with the quality of the communication with your GP during the consultation?’
Medication changes	Changes in the patient’s medication made during the consultation were observed from the transcribed audio recordings. Every decision that was made during the consultation to change something in the patient’s medication (e.g., lowering dosage, stopping, switching to a different type of medication) was noted. These reported changes were then verified using data from the GP’s electronic medical record to check if the change was indeed written down and processed in the system. This resulted in an overview of the number and types of medication changes made for each patient. Only medication changes relevant for lowering a patient’s fall risk were recorded. NB: For 6 patients, we did not have access to the data from the GP’s electronic medical record. For these patients, the medication changes were solely based on the audio recordings
Covariates	Participants provided their sex (male, female, other) and their current age (in years). Level of education was measured with a multiple-choice question and then categorized as low (no education and primary education), middle (secondary educational attainments), and high (tertiary educational attainments). Internet skills were measured with a self-reported grade ranging from 1 to 10. Health literacy was measured using all 10 items about functional health literacy, communicative health literacy, and critical health literacy from the All Aspects of Health Literacy Scale^41^. All items had three answering options: (1) seldom or never, (2) sometimes, (3) often or always, and were transformed into one mean score.

*SDM* shared decision-making, *GP* general practitioner.

### Primary outcome

The primary outcome of this study was the level of SDM between the patient and the GP. SDM was observed by using an adapted version of the Observer OPTIONMCC^38^. The Observer OPTIONMCC was developed to assess SDM between healthcare professionals and older adults with multiple chronic conditions. We created an adapted codebook, specifying for each item what it entails in the context of medication optimization related to patients’ fall risk in primary care. For each consultation, both the GP and the patient received a score ranging from 0 to 100. All consultations were assessed by two coders. One of the coders was blinded (JB), the other (LW) was not. An intraclass correlation coefficient (ICC) was used to assess the inter-observer agreement of the SDM codes. Afterward, all codes were also discussed, and any disagreement was resolved through discussion.

### Secondary outcomes

Secondary outcomes in this study were the patient-reported outcomes *decisional conflict* (measured at T2 with the decisional conflict scale, score between 0 and 100)^30^, *beliefs about medication* (measured at T0 and T2 with the beliefs about medicines questionnaire, score between −20 and 20)^39^, and *satisfaction with the quality of communication* (measured at T2 with a patient-reported grade between 1 and 10). Finally, medication changes were also assessed as a secondary outcome. These were derived from the transcribed audio recordings and verified using the data retrieved from the GPs’ electronic medical records.

### Covariates

Furthermore, several background variables were measured and controlled for in order to diminish possible confounding effects. These variables are: sex, age, level of education, internet skills, and health literacy skills.

### Statistical analyses

A power analysis revealed that sample sizes of 42 patients in both the intervention group and in the control group (3 general practices with 14 patients in each condition) yields 80% power to detect an absolute increase of 0.25 in the proportion of patients who discontinued one or more FRIDs (or received a lower dose). We assumed the proportion of patients who would stop using one or more FRIDs to be 0.05 in the control group, increasing to 0.30 in the intervention group^40^; a 0.25 difference that we considered clinically relevant. These calculations were based on a two-sided significance level of 5% and an intraclass correlation coefficient (ICC) of 0.015 to account for clustering within practices. Cronbach’s alphas were calculated to assess the internal reliability of the outcome variables measured with multicomponent instruments. An ICC was used to assess the inter-observer agreement of the SDM codes, which was summarized with Cronbach’s alpha. Scores above 0.40, 0.60, and 0.80, respectively, indicate fair, moderate, and good agreement. Data analyses were conducted according to an intention-to-treat approach. We know that all GPs in the intervention condition used the CDSS. However, we were unable to check how thoroughly they read the advice and whether they read the filled-out QPL for each patient.

Due to the clustered nature of the RCT, generalized estimating equations (GEE) analyses were performed. For the four outcome variables SDM, satisfaction with the communication, decisional conflict, and beliefs about medication, a linear GEE analysis was performed with condition (intervention or control) as the independent variable, and age, sex, health literacy, level of education, and internet skills as control variables. For the outcome measure of medication changes, two analyses were performed. For each patient, it was assessed whether at least one medication change was made (a binary variable). To assess whether this differed between the control versus intervention conditions, a binary logistic GEE analysis was performed with condition (intervention or control) as the independent variable, and age, sex, health literacy, level of education, and internet skills as control variables. Second, the total number of medication changes made was assessed for each patient (a count variable). To assess whether this differed between the control versus intervention condition, a Poisson log-linear GEE analysis was performed, again with condition (intervention or control) as the independent variable, and age, sex, health literacy, level of education, and internet skills as control variables. All analyses were conducted with IBM SPSS Statistics 29.0.

## Supplementary information

Supplementary file - CONSORT checklist

## Data Availability

The data that support the findings of this study are available on request from the corresponding author, L.W. The data are not publicly available due to the sensitive nature of the patient data.
